# Altered pattern of brain dopamine synthesis in male adolescents with attention deficit hyperactivity disorder

**DOI:** 10.1186/1744-9081-2-40

**Published:** 2006-12-04

**Authors:** Hans Forssberg, Elisabeth Fernell, Susanna Waters, Nicholas Waters, Joakim Tedroff

**Affiliations:** 1 Neuropediatrics, Department of Women and Child Health, Karolinska Institutet, Stockholm, Sweden; 2 Carlsson Research AB, Arvid Wallgrens backe 20, 413 46, Göteborg, Sweden

## Abstract

**Background:**

Limited data from positron emission tomography (PET) studies of subjects with attention-deficit/hyperactivity disorder (ADHD) indicate alterations in brain dopamine neurotransmission. However, these studies have used conventional univariate approaches that are less sensitive to detect complex interactions that may exist between different brain dopamine pathways and individual symptoms of ADHD. We aimed to investigate these potential interactions in adolescents with ADHD.

**Methods:**

We used a 3D PET scan to measure utilization of native L-[^11^C]-DOPA to map dopamine presynaptic function in various cortical, striatal and midbrain regions in a group of 8 male adolescents with ADHD and 6 age matched controls. To evaluate the interactions between the studied brain regions, multivariate statistical methods were used.

**Results:**

Abnormal dopaminergic function was found in multiple brain regions of patients with ADHD. A main finding was lower L-[^11^C]-DOPA utilization in adolescent with ADHD as compared to control subjects, especially in subcortical regions. This pattern of dopaminergic activity was correlated specifically with symptoms of inattention.

**Conclusion:**

Dopamine signalling in the brain plays an important modulatory role in a variety of motor and cognitive functions. We have identified region-specific functional abnormalities in dopaminergic function, which may help better account for the symptoms of ADHD.

## Background

Attention deficit hyperactivity disorder (ADHD) is a common neurodevelopmental disorder characterized by difficulties associated with attention, motor hyperactivity and impulsivity. The inappropriate behaviour is related to deficits of executive functions including working memory, response inhibition and motor timing [1-7]. Neural circuits in the frontal cortex and in the basal ganglia, innervated by dopamine neurons originating from the midbrain, are involved in the control of these executive functions. A large number of studies have shown that robust doses of psychostimulants reduce the symptoms of ADHD in about 80% of those prescribed the drugs [8]. Psychostimulants also enhance the performance of executive functions in subjects with ADHD [9,10] as well as in typically developed volunteers [11]. The psychostimulants methylphenidate and amphetamine increase the endogenously produced synaptic dopamine concentration through inhibition of the dopamine transporter (DAT), which takes up the dopamine into the presynaptic neurons [12]. Hence, converging evidence suggests that the behavioural problems associated with ADHD are related to cognitive dysfunctions and early disturbances of the dopamine system innervating the basal ganglia and the frontal lobe. Genetic and molecular studies have also demonstrated an association between dopamine related genes (e.g., dopamine transporter DAT1; dopamine D1 receptor; dopamine D5 receptor; dopamine D4 receptor) and ADHD [13-18].

The dopamine metabolism in the brain is a function of the presynaptic synthesis, release and reuptake of dopamine. Dopamine is synthesized from the amino acid tyrosine, which is first converted to L-dihydroxyphenylalanine (L-DOPA), and then to dopamine by the enzyme DOPA decarboxylase. The synthesis has been studied in vivo in subjects with ADHD with [^18^F]fluorodopa in two PET studies [19,20]. In the first of these studies, the authors found that adult subjects with ADHD had reduced levels of [^18^F]fluorodopa in the prefrontal cortex in comparison with control subjects. In a second study, they instead reported an increased level of [^18^F]fluorodopa in the right midbrain in adolescents with ADHD. Three independent groups, using SPECT, have reported increased density of the dopamine transporter (DAT) in the striatum in adults and children with ADHD [21-23]. However, these findings have not been replicated by other groups reporting unaltered DAT binding in the striatum [24] and lower DAT binding in the midbrain [25]. Hence, previous PET and SPECT studies indicate that there is an abnormal dopamine metabolism in subjects with ADHD, although the nature of the disturbances remains to be clarified.

In the present study we hypothesized that the behavioural problems of subjects with ADHD are associated with altered dopamine synthesis. We used the radio L-[^11^C]DOPA, (chemically identical to native L-DOPA), which is transported into the presynaptic neurons. There it is converted by the enzyme DOPA decarboxylase to L-[^11^C]dopamine and stored in storage vesicles. Therefore, PET data obtained by L-[^11^C]DOPA reflect DOPA decarboxylase activity and dopamine storage processes. The properties of the radiotracer L-[^11^C]DOPA differ in several important aspects from its more commonly used fluorine labelled analogue [26]. The turnover rate of L-[^11^C]DOPA is state-dependently modulated by drugs that affect dopaminergic systems and is thus also a measure of the functional state of the dopaminergic neurons [27-29].

The complicated interactions between different parts of the dopamine system of the brain, and disparity between the results from the existing PET studies on ADHD, indicate that straight forward comparisons between individual brain areas would not be sufficiently sensitive to detect small abnormalities in the dopamine system. Instead, we wanted to use the statistical techniques of principal component analysis (PCA) and partial least squares regression (PLS) that allow the examination of multiple brain regions simultaneously. This statistical technique has a potential value in detecting correlated changes in different areas of the brain that normally are not possible using univariate approaches. Hence, the method of applying the radiotracer L-[^11^C]DOPA for multivariate analysis have many potential applications, such as in the understanding of drug effects [30]or, as in the present investigation, to study interaction patterns within the complex dopamine systems in health and disease.

## Methods

### Participants

The study was approved by the Ethics Committee and the Radioisotope Committee of Uppsala University Hospital. Participation in the study was voluntary, and the subjects and their parents agreed to participate after receiving detailed information about the project and the methodology. Informed consent was obtained from the subjects or their parents in accordance with the Declaration of Helsinki.

#### Study group

The study group included 8 adolescent boys with ADHD 14–15 years of age. They were recruited in cooperation with child neurologists/psychiatrists at university hospitals in the county of Stockholm. Inclusion criteria were a clinical diagnosis of ADHD combined type (DSM-IV; [31], no other neurological condition, and no mental retardation. Before inclusion, a paediatric neurologist (EF) interviewed the patients and the parents, confirmed the diagnosis and rated the severity of symptoms according to DSM-IV criteria. The children's attention and hyperactivity/impulsivity scores (based on the DSM-IV criteria: 0 indicate no problem; 1 minor, 2 moderate, 3 severe problem; max = 3 × 9 = 27 in the attention and hyperactive/impulsive domain, respectively) are presented in Table 1). All participants had been tested using WISC-R [32], conventional test conducted to examine the cognitive level and cognitive profile. All had a full scale IQ of above 85. One subject had Tourette's syndrome and another one had dyslexia in addition to the ADHD. All subjects had been treated with methylphenidate in doses between 0.1 and 0.6 mg/kg/day (Table 1). One subject had finished medication and was not being treated at the time of the PET study. No other drugs were used. Importantly, all ADHD subjects were removed from medication one week prior to the PET investigation.

**Table 1:** Clinical data of the 8 adolescent boys in the ADHD group.

Subjects	Attention score	Ha/imp score	Medication	Dose mg/kg	Age (years) at start of treatment	Age (years) at PET
1	19	14	Mph	0.6	14	15
2	24	25	Mph	0.3	14	15
3	22	13	Mph	0.1	15	15
4	25	22	Mph	0.4	13	14
5	15	21	-	-	-	14
6	26	17	Mph SR	0.3	14	14
7*	9	25	Mph SR	0.3	10	15
8	19	21	Mph SR	0.3	9	15

Scores for attention and hyperactivity/impulsivity, respectively, were determined according to DSM-IV (range 0–27 for both). Type, dose and start of the psycho stimulant treatment are listed (Mph = methylphenidate, SR = slow release), as well as the age when the PET scan was performed.

#### Comparison group

Six boys 14–16 years of age, without ADHD or any other neurodevelopmental disorders constituted the comparison group.

### Tracer chemistry

The synthesis of L-[^11^C]DOPA was performed according to the standardized operational procedures at the Uppsala University PET Centre [33]. The average injected radioactive dose was 306 ± 55 MBq. The radiochemical purity was 95 ± 1% and the dose injected was about 9 μg.

### PET scanning procedure

The PET (positron emission tomography) scans were performed at Uppsala University PET Centre with a Siemens ECAT EXACT HR Plus camera (Siemens Medical Systems, Knoxville, TN, U.S.A.). The scanning program operated in 3D mode providing 15 continuous slices with a 5 mm slice thickness and with a spatial resolution of about 5 mm after image reconstruction [34]. The subjects were positioned in the PET scan with the head gently fixed in place using a foam head holder that was used for all scans. The camera was tilted to ensure that the plane of the middle image corresponded to a line 10 mm below the orbitomeatal (OM) line. Before the tracers were injected, a 10-min transmission scan was performed using an external ^68^Ga source. To decrease peripheral decarboxylation of the radiotracer subjects were pre-treated with 100 mg carbidopa one hour before the tracer injection. The scanning was started at the same time as the injection of radiotracer took place (5 ml normal saline infused over 10 s) and continued for 59 min.

The technical details used for the scanning were as follows: the frame length sequence was 3 × 60 s, 3 × 120 s, 3 × 180 s, 5 × 300 s and, finally, 2 × 480 s. Before reconstruction, the files were attenuation-corrected using data from the transmission scan, after correction for scattered radiation, and then filtered. The matrix size was 128 × 128 and the pixel size was 2.0 mm.

### Image analysis

Volume-of-distribution images (VD) were reconstructed from emission data collected 29–59 min after giving the tracer injection. A standardized ROI (region of interest) template that was created in-house using MRI (magnetic resonance imaging) was overlaid on the VD images for each subject and then manually adjusted to correct for anatomical differences [30].

The 28 regions included in the ROI template are in the list of abbreviations. The midbrain section was located in the mesencephalon in the region of the substantia nigra and ventral tegmentum. A ROI in the occipital cortex was used as reference because this region is devoid of dopamine synthesis. Computer processing was used to determine the rate of dopamine synthesis by taking the time activity curve for the occipital cortex, and generating images of the influx rate, known as Ki images, by performing a weighted linear regression using a two compartment mathematical description for the process [35]. As a result, a value for the unidirectional influx of radioactivity (i.e., dopamine synthesis) was generated for each pixel in a Ki image. Thus, the Ki values relate to the *in vivo *synthesis of dopamine in presynaptic neurons in the brain. The dopamine synthesis and release was studied in cortical, striatal and midbrain regions.

### Statistical analyses

A PET scan of the brain generates a massive amount of data. Traditionally such data has been analyzed using univariate approaches, however, such methods cannot take into account the complexity of the dopamine system or allow specific profiles of different brain regions to be made and the interactions between them. The data set, comprised of L-[^11^C]DOPA Ki values, required further analysis to determine whether there were indeed differences between the adolescents with and without ADHD. Thus, the data sets were explored using the statistical technique partial least squares regression (PLS), which is closely related to principal components analysis (PCA) [36,37]. These techniques are generally useful to explore biological data, which typically display considerable noise and co-linearity's making the use of e.g. multiple regression or univariate analysis less adequate. Accordingly, they have widespread use in e.g. imaging and medicinal chemistry. In essence, PCA and PLS utilizes the co-variances present among the variables to create a compact and noise-reduced representation of the data. In PLS, which is used here, one ore more dependent variables are selected in order to create regression models describing the relationships between the dependent vs. the independent variables. This procedure yields regression coefficients which are analogous to the coefficients calculated in e.g. multiple regressions. The statistical significance was assessed using the cross-validation procedure. This procedure rejects models that are strongly influenced by outlying observations, i.e., such models are not considered significant. All multivariate calculations were performed using the Simca-P 10 software (Umetrics Inc., USA). Standard errors of the regression coefficients were calculated using the Jack-knife procedure [38]. For average analysis of regional Ki values STATISTICA for Windows was used [39]. The Sheffe F-test was used for post hoc testing. The level of significance was set to *P *< 0.05.

## Results

**Figure 1:**
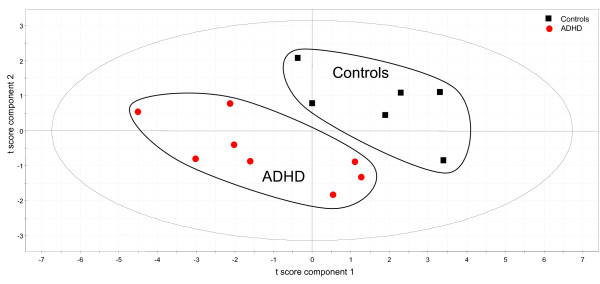
Partial least squares regression (PLS) discriminant analysis distinguishes ADHD patients (red circles) from control subjects (black squares). Each object in the graph represents one individual. For each individual, the position in the plot is a compact representation of all regional K_i _data collected. Objects that appear close to each other are generally similar across all variables. In this case, ADHD patients and controls form separate groups, reflecting underlying group differences in regional K_i _values.

The ADHD subjects displayed lower Ki values in most regions than the control group (Table 2). However, in an Anova analysis, only the midbrain section showed a significant decrease compared to the controls. Since the Anova approach is not ideal for the analysis of several simultaneous measurements, the dataset was further analyzed using multivariate analysis. To compare the ADHD and control groups, PLS analysis was performed with a discriminant variable incorporated denoting a diagnosis of having ADHD or of not having ADHD. The analysis yielded a statistically significant one-component model separating the ADHD subjects from the controls (R2X = 0,27, R2Y = 0.43, Q2 = 0.081). The subjects are clearly distributed in two groups (Figure 1).

**Table 2:** Regional L-[^11^C]DOPA influx rate (Ki*10) in the 28 regions included in the ROI template (see list of abbreviations) in the ADHD and control groups.

Region	Controls		ADHD		Diff (%)	
	r	l	r	l		
Caudd	1,62	1,60	1,50	1,47	-7,3	-8,0
Caudint	1,67	1,65	1,64	1,61	-1,8	-2,7
Caudv	1,76	1,77	1,66	1,65	-5,4	-6,6
Putd	1,36	1,49	1,48	1,55	8,6	3,4
Putint	1,87	1,88	1,67	1,82	-10,7	-3,0
Putv	1,93	1,87	1,73	1,73	-10,4	-7,2
Mpfd	0,66	0,61	0,61	0,63	-8,0	4,2
Mpfi	0,64	0,60	0,61	0,63	-5,4	5,5
Mpfv	0,64	0,63	0,61	0,56	-4,7	-10,0
Pfd	0,46	0,45	0,42	0,45	-8,1	-0,3
Pfi	0,46	0,45	0,42	0,45	-9,9	-0,3
Pfv	0,44	0,48	0,47	0,47	6,5	-2,4
Acc	1,77	1,81	1,54	1,61	-12,7	-11,0
Midbrain	0,90	0,88	0,75*	0,66*	-17,2	-24,9

The difference between the groups for each region is expressed in percentage. Only midbrain values reached statistical significance (ANOVA).

**Figure 2:**
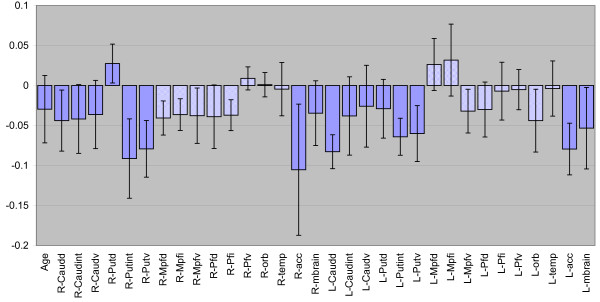
Regression coefficients in the PLS model discriminating ADHD patients vs. control subjects. A large positive coefficient indicates an increase in the corresponding variable in ADHD patients vs. controls. In this model, most coefficients are negative, reflecting a general decrease in K_i _values in the ADHD group. In particular, subcortical K_i _values (dark blue columns) tend to be negatively related to the ADHD diagnosis. See list of abbreviations to identify the anatomical ROIs

The PLS regression coefficient indicates which variables are most responsible for the separation of the two groups (Figure 2). Such coefficients are interpreted in the same way as in ordinary regression analysis and, consequently, positive coefficients indicate positive correlations and the size of the coefficient reflects the strength of the correlation. In this case, the dependent variable is a discriminant variable denoting the ADHD diagnosis, so that positive coefficients indicate increases in comparison with the control group, and negative coefficients indicate decreases. Thus, the dominance of negative columns in Figure 2 shows that ADHD subjects generally have lower Ki. Furthermore, the regression coefficients suggest that predominantly subcortical Ki values (dark blue) are low, while cortical Ki values (light blue) are less affected.

**Figure 3:**
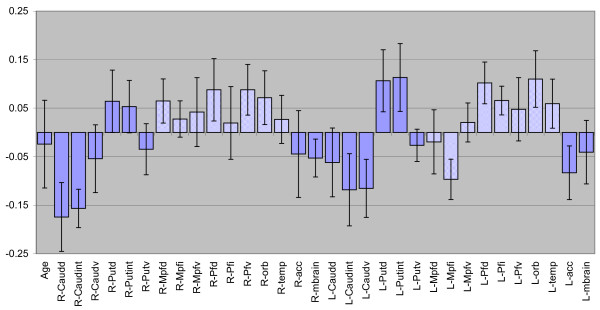
Regression coefficients in the PLS model relating attention score to regional Ki values in the ADHD group only. Large positive coefficients indicate a positive correlation, i.e., the larger the Ki, the higher the attention score. Large negative coefficients suggest an inverse relationship, where high Ki values are associated with low values in the attention score.

To further explore the relationship between the Ki indices and ADHD symptoms, a PLS regression model was created. This describes the dependence of the *attention *score (obtained using DSM-IV) on the Ki indices in the ADHD patients. The attention scores were taken as the Y variables, and Ki values as the X variables. One patient with a remarkably low attention (an outlier) who exhibited a good response to methylphenidate was excluded from this regression model (see patient 7; Table 1). The model was statistically significant, indicating a strong relation between Ki and attention (R^2^X_cum _= 0.46, R^2^Y_cum _= 0.982, Q^2^_cum _= 0.726 for the two first components). The R2Ycum statistic is analogous to the coefficient of determination (R2) used in correlation analysis. In this model, R2Y is large, 98%, reflecting a strong correlation between the Ki and the attention score data. The relations between Ki values and attention are further visualized in Figure 3, where the PLS regression coefficients are shown. Most cortical variables tend to be positively related to attention, whereas the subcortical Ki indices, with few exceptions, are negatively correlated to attention. Thus, low subcortical Ki values are associated both with the ADHD diagnosis per se, as demonstrated by the first PLS model (Figure 2), and with more severe attention deficits within the ADHD group (Figure 3). Cortical variables appear to be less important with respect to group discrimination, but have a positive correlation to attention within the ADHD group. When patient 7 with the deviating score was included, the model showed the similar trend, but did not reach significance due to the increased variation in the small sample (n = 8).

An attempt to model the *hyperactivity/impulsivity *score (DSM-IV) in relation to regional Ki values in a similar way failed to produce a significant model. Thus, we find no association between regional dopamine synthesis and hyperactivity/impulsivity. With the data at hand we cannot determine whether this is due to a true lack of association, or if e.g. a large noise level in the hyperactivity/impulsivity scores, or heterogeneity in the ADHD population, obscures the relationship.

## Discussion

Bearing in mind the limitations of this study (see below), we have shown that it is possible to distinguish ADHD subjects from age matched controls by applying multivariate statistical methods to regional L-[^11^C]DOPA Ki values. In most brain areas in the subjects with ADHD the rate of dopamine synthesis was lower than in the healthy controls, with the values being particularly low in the subcortical regions. The low synthesis in the subcortical areas correlates with the severity of the attention deficit symptoms, assessed according to the DSM-IV. These findings are in good agreement with extensive experimental and clinical research supporting the notion that dopamine system plays a modulatory role in attention processes, while impulse control is more linked to other neurotransmitter systems, e.g., serotonin [40]

There are several limitations of this study. Most of our subjects with ADHD were not drug naïve, and although all were medication-free for at least 1 week, the washout period may not have been sufficient to reset the dopamine system to a basal level. In addition, psychostimulants are known to produce long term effects, e.g., dendrite growth in prefrontal cortex of rodents [41] and down-regulation of the dopamine D2 receptor in primates [42]. Therefore, at this point we cannot exclude any effects of the medication and therefore new studies need to be performed on drug naïve subjects. Moreover, two of the subjects with ADHD had additional neurological disorders. In particular, the subject with Tourette's syndrome could be a confounder since this condition is associated with abnormal dopamine metabolism [43]. However, it should be noted that the method used to establish statistical significance of the multivariate models, cross-validation, is based on the calculation of how stable the results are as different subjects are excluded. Models in which the relationships observed rely on "outliers" will therefore not be considered statistically significant by the cross-validation criterion. Exclusion of the two comorbid cases did not affect the results. Hence, although the sample size is small and includes individuals with comorbid conditions, the applications of multivariate statistical methods are able to demonstrate significant results.

The low L [^11^C]DOPA Ki values suggest that children with ADHD differ from controls in their metabolism of DOPA over a range of cortical and subcortical regions. It is most likely that the low values reflect aberrations in the presynaptic synthesis and release of dopamine. These results support earlier theories that a hypofunction of the dopamine system underlies the behavioural symptoms in ADHD [44]. This theory was based on the positive effects of stimulant drugs in children with ADHD and that such drugs facilitate the endogenous monoamine transmission by blocking the dopamine transporter. Later, Volkow and co-workers have in a series of elegant PET studies shown that methylphenidate increases the endogenous level of dopamine and that it interacts with mental tasks triggering dopamine release [45,46]. However, there has been little support for lower dopamine synthesis in previous SPECT and PET studies in subjects with ADHD. PET studies on the uptake of [^18^F]fluorodopa have demonstrated that there is a *decreased *uptake in the frontal cortex and an *increased *uptake in the midbrain [19,20]. The latter result is opposite to ours, obtained by studying the uptake of L-[^11^C]DOPA. The differences might be attributable to the different specificity of the radioligands. As a radiotracer, L-[^11^C]DOPA seems to detect changes in the functional tone in the dopaminergic systems more readily than its fluorine labelled analogue [26].

Other SPECT and PET studies of dopamine transporter binding in ADHD have reported increased binding capacity in the striatum of adults and children with ADHD [21-23,47]. However, this result was not replicated by other groups, who instead reported either an unaltered or a reduced DAT- binding [24,48]. In a recent PET study on adolescent boys with ADHD, we used a newly developed cocaine analogue radioligand, [^11^C]PE2I, that binds selectively to the dopamine transporter [25,49]. We could not confirm the first SPECT studies indicating an increased DAT binding in striatum, but rather obtained results more closely in agreement with the studies indicating an unaltered or decreased binding capacity. In addition, the high performance of 3D PET and the good characteristics of the new radioligand allowed us to detect a clear reduction of the DAT binding in the midbrain. These two recent studies from our group, indicating a lower dopamine synthesis and a reduced density of DAT in the midbrain, correspond well to the earlier theories of reduced dopamine signalling [44]. From our results it is not possible to determine the primary cause of the imbalance. A reduction of the presynaptic dopamine synthesis and release could be compensated for by decreased DAT activity. Genetic studies in subjects with ADHD have identified polymorphism in several specific genes that encode components of catecholamine signalling system, among them polymorphism in the DAT gene [13]. In some subjects, a lower uptake of dopamine from the synaptic cleft may thus be the primary cause. This could subsequently induce secondary adaptations of the dopamine release and synthesis via presynaptic autoreceptors [50]. Recently, in an animal model of ADHD (using the spontaneously hypertensive rat), the expression of specific genes involved in dopamine neuron differentiation and functioning during the postnatal development of the midbrain was studied [51]. The data showed transient reductions of the expression of tyrosine hydroxylase and dopamine transporter genes, suggesting a down-regulation of the dopamine transmission at this stage of development, which is in line with the present study and which supports the hypodopaminergic hypothesis of ADHD.

In the present study, ADHD subjects tended to display lower Ki values over most regions. However, with closer examination, a *region-specific *pattern emerges. The most profound decreases are seen in subcortical areas (Figure 2 and Table 1), such as the nucleus accumbens, putamen and the midbrain, while cortical areas show smaller decreases, and even a tendency towards increases in some prefrontal areas. This indicates that there are regional differences and that the ADHD specific alteration of the dopamine synthesis is region specific. The possibility of different dopamine abnormalities in different brain regions in ADHD has been discussed previously by, e.g., Castellanos [52], who suggested that there was a difference between areas innervated by the two dopamine pathways originating from the substantia nigra and the ventral tegmental area, respectively. The motor hyperactivity and impulsivity could be caused by overactivity in the basal ganglia, innervated by the substantia nigra, while the inattention could be due to an underactivity in the cortical areas innervated by the ventral tegmentum. Our data corresponds with this hypothesis to a certain extent, i.e., our data indicate that the DOPA metabolism in the basal ganglia seems to have a different pattern to that in the cortical areas. However, our data on the DOPA metabolism is hard to translate into terms of increased or reduced activity in the neural networks controlling the functional activity because the dopamine may have both facilitatory and inhibitory effects, depending on, e.g., the profile of the local dopamine receptor population on the postsynaptic neurons [53].

There was a strong relationship between attention and the regional Ki values within the ADHD Group. First, the results indicate that the Ki pattern not only discriminates between the ADHD group and the controls, but that it is also related to the severity of the attention difficulties of the individuals with ADHD. Most subcortical Ki values are inversely related to attention, i.e., the lower the Ki values the more problems the subject has with inattention. Conversely, in several cortical regions the Ki are positively related to the severity of symptoms. Again, it appears that the subcortical and cortical DA systems are differentially affected in patients with ADHD and that the resulting behavioural effects differ.

Most PET and SPECT studies on ADHD subjects have so far indicated that there are disturbances both in the pre- and postsynaptic dopamine transmission, but the results have been inconsistent. The reasons for these discrepancies probably reflect the use of different imaging techniques (PET and SPECT) and different radioligands, as well as differences in the age and medication history of the populations studied. A fundamental problem exists in addition to this, which is that the ADHD construct is based on behavioural symptoms and therefore includes heterogeneous populations of children with different cognitive and behavioural dysfunctions. This functional variation is probably associated with certain molecular variations in specific brain areas that may be large in relation to a more subtle general difference between ADHD subjects and controls. In the work reported here, it was only possible to reveal a general pathological pattern of the complex dopamine system that is shared by subjects with core ADHD symptoms by conducting multivariate analysis on data obtained by PET.

## Conclusion

Attempts to create a single theory explaining ADHD have failed so far. This is not remarkable in view of the present study, revealing the complexity of the interaction between the dopamine synthesis in the various subcortical and cortical areas and between this interactive pattern and the symptoms of attention deficit. We have only studied one component in the complex chain of dopamine signalling. It is obvious that the neurobiological mechanisms underlying ADHD are numerous and interactive. The challenge for future research is to characterize various subtypes of ADHD and, in particular, to determine the underlying cognitive dysfunctions and their interaction with various neural and neurotransmitter systems.

## Abbreviations

*R-Caudd*, *R-Caudint*, *R-Caudv *are the dorsal, intermediate and ventral sections of the right caudate nucleus; R-*Putd*, *R-Putint*, *R-Putd *are the dorsal, intermediate and ventral sections of right putamen; R-*Mpfd*, *R-Mpfi*, *R-Mpfv *are the dorsal, intermediate and ventral sections of the right mesial prefrontal cortex; R-*Pfd*, *R-Pfi*, *R-Pfv *are the dorsal, intermediate and ventral sections of the right dorsolateral prefrontal cortex; R-*Acc *is the right nucleus accumbens; R-*Mbrain *is the right midbrain; the prefix *L *indicates the same areas on the left side.

## Competing interests

HF is shareholder and scientific adviser for COGMED Cognitive Medical Systems AB, which develops cognitive training programmes aimed to improve working memory function.

## Authors' contributions

HF participated in the conception, design and writing of the study. EF participated in the planning of the study recruited patients and performed the clinical assessments. SW and NW performed post processing of PET data and the statistical analyses. JW participated in the design of the study, acquisition and analyses of PET data and helped to draft the manuscript. All authors read and approved the final manuscript.
